# Ketone Body β-Hydroxy-Butyrate Sustains Progressive Motility in Capacitated Human Spermatozoa: A Possible Role in Natural Fertility

**DOI:** 10.3390/nu15071622

**Published:** 2023-03-27

**Authors:** Claudia Pappalardo, Federica Finocchi, Federica Pedrucci, Andrea Di Nisio, Alberto Ferlin, Luca De Toni, Carlo Foresta

**Affiliations:** Unit of Andrology and Reproductive Medicine, Department of Medicine, University of Padova, 35128 Padova, Italy

**Keywords:** follicular fluid, ketosis, capacitation, succinyl-CoA transferase, metabolism

## Abstract

*Background* Calorie restriction is recognized as a useful nutritional approach to improve the endocrine derangements and low fertility profile associated with increased body weight. This is particularly the case for dietary regimens involving ketosis, resulting in increased serum levels of ketone bodies such as β-hydroxy-butyrate (β-HB). In addition to serum, β-HB is detected in several biofluids and β-HB levels in the follicular fluid are strictly correlated with the reproductive outcome in infertile females. However, a possible direct role of ketone bodies on sperm function has not been addressed so far. *Methods* Semen samples were obtained from 10 normozoospermic healthy donors attending the University Andrology Unit as participants in an infertility survey programme. The effect of β-HB on cell motility in vitro was evaluated on isolated spermatozoa according to their migratory activity in a swim-up selection procedure. The effect of β-HB on spermatozoa undergone to capacitation was also assessed. *Results* Two hours of exposure to β-HB, 1 mM or 4 mM, proved to be ineffective in modifying the motility of freshly ejaculated spermatozoa isolated according to the migratory activity in a swim-up procedure (all *p* values > 0.05). Differently, sperm maintenance in 4 mM β-HB after capacitation was associated with a significantly higher percentage of sperm cells with progressive motility compared to β-HB-lacking control (respectively, 67.6 ± 3.5% vs. 55.3 ± 6.5%, *p* = 0.0158). Succinyl-CoA transferase inhibitor abolished the effect on motility exerted by β-HB, underpinning a major role for this enzyme. *Conclusion* Our results suggest a possible physiological role for β-HB that could represent an energy metabolite in support of cell motility on capacitated spermatozoa right before encountering the oocyte.

## 1. Introduction

Body mass composition and dietary regimen are claimed as major determinants of health status, particularly with regard to fertility outcomes [1]. Increased body adiposity associated with high-calorie intake and malnutrition has been frequently linked to endocrine derangements and infertility in both males and females [2,3]. On the other hand, weight loss and appropriate nutritional regimens are considered first-line strategies to improve all the aforementioned clinical conditions [4,5]. Among the most effective dietary regimens, those involving calorie restriction and an increase in blood ketone-bodies levels, or “ketosis”, are currently gaining a great deal of consensus. From a biochemical point of view, ketosis arises when the calorie intake shifts from largely based on carbohydrates, namely ~60% of the total calorie intake, to almost exclusively based on fatty and protein substrates [6]. This condition triggers massive fatty acid β-oxidation to support body energy demand, with the generation of ketone-bodies as specific by-products with the role of energy intermediates, such as aceto-acetic acid and D-3-hydroxy-n-butyric acid, also known as β-hydroxy-butyrate (β-HB) [7]. On the other hand, from a nutritional point of view, ketosis can be obtained through different dietary approaches. Prolonged fasting is a condition associated with the shift from carbohydrate to fat catabolism as the primary energy source and results in a significant increase in serum β-HB levels greater than 0.5 mmol/L [8]. Moreover, a standard body weight-based normocaloric diet characterized by 70% fat, 20% protein, and only 10% carbs is sufficient to rapidly increase ketone-bodies levels in humans and is thus called the ketogenic diet [9]. Importantly, the normocaloric-ketogenic diet is an effective non-pharmacological treatment for epilepsy in children, especially in those showing drug-resistant epileptic states [10]. With the clinical aim of body-weight control, the aforementioned ketogenic diet composition is frequently associated with various degrees of calorie restriction and is thus called low calories- or very low calories-ketogenic diet. In quantitative terms, as recently addressed by a meta-analysis from Furini et al., the weight reduction after the application of normo-calories or very low calories-ketogenic diets reported as a standardized mean difference, is, respectively, 1.05 and 3.25 [11].

In males, a strict correlation between overweight-obesity and hypogonadism has been widely reported [12,13]. However, a clear role of weight loss dietary strategies, and specifically of the ketogenic diet, on the improvement of testosterone levels is currently a matter of debate [11,14]. In addition to the possible retrieving of the proper endocrine pattern through weight lowering, other additional or parallel mechanisms on the testis function associated with nutritional-based ketosis, including fertility outcomes, are largely underinvestigated [6,15]. The biofluid profile of low/mid molecular weight metabolites has recently become a promising research field for the pathophysiological biomarkers of organ function [16]. This is particularly the case of human semen in which the analysis of metabolite composition showed interesting correlations with fertility outcomes that are currently uncovered by classical morphology-based semen analysis [17].

However, to date, there is no evidence describing either the detection or the concentration of ketone bodies in human semen. Differently, a recent study that focused on the metabolomic profile of follicular fluid (FF) in relation to fertility outcome in women showed a significant reduction in β-HB in the FF from patients with recognized causes of infertility such as polycystic ovary syndrome or endometriosis [18]. Since spermatozoa enter into contact with the follicular fluid during the latest stages of fertilization of the oocyte, this evidence suggests a possible direct role of β-HB on sperm function.

On these bases, in this study we evaluated the effect of β-HB on sperm motility parameters in ejaculated human spermatozoa at basal and after the capacitation procedure, supporting its role as an energy substrate in the very late phase of the very last stages of cell migration across the female genital tract.

## 2. Materials and Methods

### 2.1. Chemicals

Sodium β-hydroxy-butyrate (cat# 54965) and succinyl-CoA transferase (SCOT) inhibitor aceto-hydroxamic acid (AHX-A, cat# 159034) and albumin from human serum-fatty acids free (cat# A3782) were all purchased from Merck-Sigma-Aldrich (Milan, Italy). A sperm-washing medium (SWM) was purchased from Fujifilm-Irvine Scientific Inc. (Buccinasco (MI) Milano, Italy).

### 2.2. Semen Samples

The study was approved by the Institutional Ethics Committee of the University Hospital of Padova, Italy (protocol number 2208P and successive amendments). All subjects provided signed informed consent for the study, which was performed according to the principles of the Declaration of Helsinki. Semen was obtained from 10 normozoospermic healthy donors attending the University Andrology Unit as participants in an infertility survey programme (mean age 22.1 ± 3.7 years).

In order to reduce any inter-subject variability, patients underwent 4 separate sessions of semen donation by masturbation, taking care to maintain at least 3 days of sexual abstinence between each donation. Samples were allowed to liquefy for 30 min at 37 °C and then examined according to the World Health Organization criteria for human semen analysis [19]. The motile sperm fraction was isolated by the swim-up procedure as previously described [20]. Briefly, liquified semen was placed in a test tube and fresh SWM was stratified on top of the semen. The progressive motile sperm fraction was allowed to migrate toward the SWM for 45 min at 37 °C and then isolated from low and immotile cells left in the semen residue. Motility parameters were evaluated by a Sperm Class Analyzer (CASA System, Hamilton Thorne Inc., Beverly, MA, USA), including average path velocity (VAP, in μm/s), straight-line (rectilinear) velocity (VSL, in μm/s), sperm curvilinear velocity (VLC, in μm/s), beat-cross frequency (BFC, in Hz), straightness (STR as VSL/VAP, in %), and linearity (LIN, in %). Sperm capacitation was induced by the incubation of spermatozoa with Biggers, Whitten, and Whittingham medium (BWW), supplemented with 25 mEq/L of sodium bicarbonate and with 3.5% of human serum albumin, for two hours at 37 °C as previously described [21]. β-HB and AHX-A were then added to the capacitating medium at the concentrations indicated below and the sperm progressive motility was monitored for the following two h.

## 3. Results

**Figure 1:**
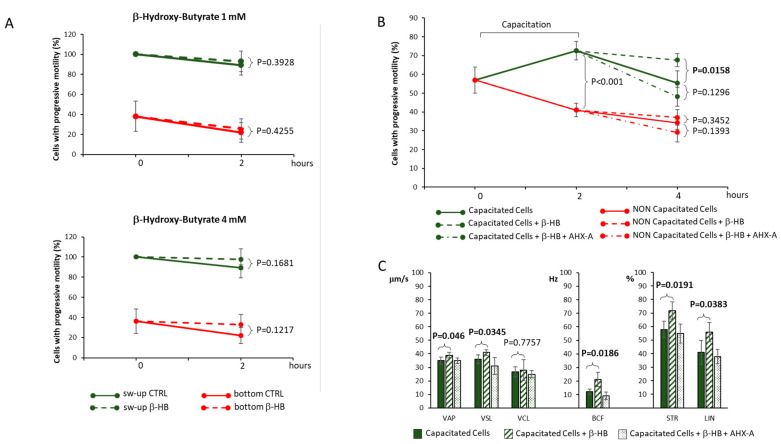
(**A**) Effect of 2 h exposure to 1 mM or 4 mM β-hydroxy-butyrate (β-HB, dashed lines), on cell progressive motility, in sperm cells from normozoospermic subjects, isolated according to their highly migratory (sw-up, green lines) or low migratory (bottom, red lines) activity in a swim-up procedure. Continuous lines refer to β-HB-lacking controls. Results show the mean values of 4 independent experiments. (**B**) Effect of 2 h exposure to 4 mM β-HB (dashed lines) on cell progressive motility, in sperm cells undergone to capacitation (green lines) or non-capacitated controls (red lines). Point line traits refer to experimental conditions involving exposure to β-HB and the succinyl-CoA transferase inhibitor aceto-hydroxamic acid (AHX-A). (**C**) Sperm motility parameters, evaluated with a Sperm Class Analyzer (CASA), in capacitated sperm cells maintained for 2 h in capacitating medium, in capacitating medium with the addition of 4 mM β-HB, or in capacitating medium with the addition of 4 mM β-HB and AHX-A. CASA parameters included average path velocity (VAP), straight-line (rectilinear) velocity (VSL), sperm curvilinear velocity (VLC), beat-cross frequency (BFC), straightness (STR as VSL/VAP), and linearity (LIN). Results show the mean values of 4 independent experiments. Significance: P levels between indicated conditions are reported.

The effect of β-HB on cell motility in freshly ejaculated spermatozoa was first evaluated (Figure 1A). In particular, highly motile and poorly motile sperm fractions were obtained from normozoospermic healthy donors by standard swim-up technique as previously described [20]. The two isolated fractions were exposed for 2 h to 1 mM or 4 mM β-HB at 37 °C and the percentage of cells with progressive motility was then evaluated. Compared to control conditions, in which β-HB was omitted, exposure of both sperm populations to β-HB at either concentration was associated with a slight but not significant increase in sperm motility (Figure 1A; all *p*-values > 0.05).

Since sperm motility is recognized to be subtended by complementary signaling cascades according to the capacitation status [22], the possible effect of β-HB on capacitated spermatozoa was then evaluated (Figure 1B). To this aim, freshly-ejaculated sperm cells were washed with SWM and then subjected to the capacitation procedure for 2 h as previously described [21]. Subsequently, cells were maintained for a further 2 h in either capacitating BWW medium, serving as a control condition (CTRL), capacitating BWW medium supplemented with 4 mM β-HB or BWW medium supplemented with 4 mM β-HB and the SCOT inhibitor AHX-A at a concentration of 50 μg/mL. The same treatments were also applied to non-capacitated spermatozoa as the reference condition. As expected, capacitation was associated with a significant increase in the percentage of spermatozoa with progressive motility, compared to non-capacitated cells (72.6 ± 4.9% vs. 41 ± 3.6%, *p* < 0.001). Compared to the CTRL maintenance in BWW medium, the two h maintenance in 4 mM β-HB after capacitation was associated with a significantly higher percentage of sperm cells with progressive motility (respectively, 55.3 ± 6.5% vs. 67.6 ± 3.5%, *p* = 0.016; Figure 1B). Differently, the co-incubation of β-HB with AHX-A was associated with levels of progressive motility comparable with CTRL (48.1 ± 5.0%, *p* = 0.129 vs. capacitated CTRL). The evaluation of sperm motility parameters with the CASA System (Figure 1C) showed that compared to the sole capacitating medium (CTRL), the maintenance in 4 mM β-HB after capacitation was associated with a significant increase in VAP (CTRL 35.2 ± 2.5 μm/s vs. β-HB 39 ± 2.1 μm/s, *p* = 0.046), VSL (CTRL 35.8 ± 3.1 μm/s vs. β-HB 41 ± 1.9 μm/s, *p* = 0.0345), BFC (CTRL 11.9 ± 1.95 Hz vs. β-HB 21.0 ± 5.3 Hz, *p* = 0.0186), STR (CTRL 58.2 ± 6.1% vs. β-HB 71.8 ± 6.4%, *p* = 0.0191), and LIN (CTRL 41.0 ± 8.7% vs. β-HB 56.1 ± 7.3%, *p* = 0.0383). The co-incubation of β-HB with AHX-A was associated with the blunt of all the aforementioned parameters to levels comparable with CTRL conditions (all *p*-values > 0.05).

In non-capacitated conditions, exposure to β-HB or β-HB + AHX-A was associated with no significant variation of sperm progressive motility compared to CTRL (all *p*-values > 0.05).

## 4. Discussion

In the present study, we provide evidence that exposure to β-hydroxy-butyrate, a major ketone body, is associated with the maintenance of higher levels of progressive motility and sperm motility parameters in capacitated spermatozoa, compared to control conditions in which β-HB is absent. Importantly, this effect is blunted by the presence of a known succinyl-CoA transferase inhibitor, supporting a key role of this metabolic enzyme.

The increase in blood or urine levels of ketone bodies, namely ketosis, is a metabolic condition that takes place in course of low glucose availability, generally deriving from low-carbohydrate diets or a fasting state [23]. Ketosis, by itself, is a condition of metabolic stress which may exacerbate the clinical risk of pre-existing disease conditions such as liver, kidney, or pancreatic insufficiency [24]. However, from an evolutionary point of view, humans developed in order to “cohabit” with ketosis during their ancestral transition from essentially herbivorous to essentially carnivorous, approximately two to three million years ago [25]. In fact, it is believed that the transition to the consumption of animal proteins has favored the rapid increase in the volume and architecture of the brain [25]. The evolutionary advantage deriving from this anatomical characteristic would have been much greater than the disadvantage of periodically finding themselves in the scarcity of food linked, for example, to the seasonality of the species preyed on [25]. Aiming to compensate for the caloric restriction deriving from these periods of forced fasting, the organism would therefore have evolved to optimize the use of energy storage deposits in the adipose tissue, through the release of high-energy substrates such as fatty acids [26]. In addition, another major feature of ketone bodies is that of glucose sparing together with proteolysis prevention [27,28]. This is of particular importance in extra-hepatic tissues, such as the heart, kidney, and skeletal muscle, characterized by a wide range of energy metabolites usage and expressing the key enzyme involved in ketolysis, namely succinyl-CoA transferase, which allows the incorporation of ketone bodies into tricarboxylic acid cycle [29,30,31]. On these bases, there are no evolutionary elements that argue in favor of a negative influence of ketosis on the reproductive outcome.

Interestingly, available studies conducted in animal models showed that ketone bodies are commonly found in different biofluids where the respective levels essentially reflect changes in serum [32]. 

Despite there being no available data about the possible presence of ketone bodies in seminal plasma, the detection of β-HB in follicular fluid has been recognized since the early 2000s, especially from studies in livestock animal models, where the possible correlation between the FF profile of medium-low molecular weight metabolites and the reproductive outcome was investigated [33,34]. In humans, this evidence is rather recent and the functional correlates of β-HB levels in FF are still a matter of debate [35]. To this regard, Castiglione Morelli et al. recently showed that metabolic pathways involved in the onset of polycystic ovary syndrome and endometriosis are essentially matched with the pattern of low/mid molecular weight metabolites detected in FF, such as the increased levels of glucose and the decreased levels of acetate and β-t [18]. On the other hand, sperm cells have been recognized as expressing succinyl CoA transferase (SCOT), the key mitochondrial enzyme involved in the metabolism of ketone bodies, therefore representing potential users of β-HB [36] and the possible role of the latter as an energy substrate for spermatozoa.

In the present study, we report that the exposure of freshly ejaculated sperm cells to β-HB is poorly or essentially ineffective in modifying major sperm parameters such as cell motility, confirming early data obtained in bovine sperm models by Missio et al. [34]. Differently, we showed that β-HB was able to sustain the progressive motility over time only in sperm cells that underwent capacitation. This hypothesis is supported by detailed sperm motility parameters obtained with a computer-assisted sperm class analyzer, where the increased levels of straight-line velocity, straightness, and linearity parameters upon β-HB exposure suggest a role of this ketone body in progressive motility maintenance in capacitated sperm cells. Importantly, such an effect was likely mediated by SCOT, since it was significantly blunted by the SCOT inhibitor AHX-A. A possible interpretation of these results can be drawn in light of the chain of events that feature sperm migration through the female genital tract towards the fertilization site. In particular, dealing with sperm processes that occur in close proximity to the oocyte before its fertilisation. In this precise physiological context, only a restricted sub-population of spermatozoa is able to access the fertilization site, upon the selection operated by the motility function and by the ability to respond to appropriate chemo-, rheo- and thermo-tactical gradients that are generated in the female genital tract [37,38,39]. In addition, through the peculiar composition of the FF, which is rich in albumin and bicarbonate ions, sperm cells undergo capacitation, a complex process associated with the reduction in membrane cholesterol and alkalization of the cytoplasm [40,41]. Following these biochemical events, specific signaling pathways are triggered, such as the cAMP-dependent protein kinase A (PKA) activation, resulting in the overall increase in sperm progressive motility by spermatozoa, also known as a gain of hypermotility [37,38,39]. The fact that exposure to β-HB is associated with a slower decline in progressive motility over time only upon capacitation, on one hand, renders ketone bodies as a possible auxiliary energy supply to be used in the late end of the sperm migration. In addition, this may represent an additional screening factor for spermatozoa competent for fertilization. In fact, according to this model, only properly capacitated spermatozoa expressing the correct pattern of metabolic enzymes would be able to use β-HB released with the FF upon ovulation, as a ready-to-use energy metabolite immediately prior to fertilization [42].

While acknowledging the absolutely preliminary nature of these results, they open up some challenging hypotheses. First of all, the possible role of metabolic pathologies in modifying the interaction between the female peri-ovocytic microenvironment and male sperm function. Secondly, the possible effect of weight loss strategies, involving low- or very low-calories ketogenic diets on natural or assisted fertility, applied to female patients with infertility linked to metabolic factors.

## 5. Conclusions

In this study, we provide evidence that exposure to β-HB is associated with a lower decline of sperm progressive motility in capacitated spermatozoa. This effect is likely mediated by the key metabolic enzyme succinyl-CoA transferase, involved in ketone bodies catabolism, expressed in spermatozoa. This evidence opens questions about the possible role of the β-HB, released by the follicular fluid upon ovulation, an energy supply metabolite in the late end phases of the oocyte fertilization. Further studies are required to confirm these data and to address the cell pathway linking capacitation to SCOT activation in sperm cells.

## Data Availability

Date will be provided upon request.
